# Effects of acupoints-based TENS combined with tDCS on spasticity and motor function in ischemic stroke with spastic hemiplegia: study protocol for a randomized controlled trial

**DOI:** 10.3389/fneur.2023.1269472

**Published:** 2023-11-27

**Authors:** Xu Qian, Liang-Xiao Ma, Ling-Hui Ma, Qin-Yong Zhang, Jing-Yun Xiu, Xiu-Yan Wang, Tian-Yi Sun

**Affiliations:** ^1^School of Acupuncture-Moxibustion and Tuina, Beijing University of Chinese Medicine, Beijing, China; ^2^The Key Unit of State Administration of Traditional Chinese Medicine, Evaluation of Characteristic Acupuncture Therapy, Beijing, China

**Keywords:** acupoint-based transcutaneous electrical nerve stimulation, stimulation frequencies, transcranial direct current stimulation, ischemic stroke, spastic hemiplegia, randomized controlled trial

## Abstract

**Background:**

Spastic hemiplegia following ischemic strokes seriously impedes the recovery of motor function posing a vast rehabilitation challenge. As the uncertain effects of recommended conventional treatments such as botulinum toxin injections on active functional improvement and potential adverse effects cannot be bypassed, there is an increasing need in alternative, more effective and safer modalities. Acupoints-based transcutaneous electrical nerve stimulation (Acu-TENS) and transcranial direct current stimulation (tDCS) are effective non-invasive modalities for stroke rehabilitation, particularly showing anti-spastic effect and functional improvements as well. However, the optimal stimulation frequency of Acu-TENS and whether combination of Acu-TENS and tDCS exert synergistic effect remain to be investigated.

**Objective:**

To evaluate the effects of Acu-TENS combined with tDCS on spasticity and motor function in ischemic stroke patients with spastic hemiplegia and screen the optimal frequency of Acu-TENS.

**Methods:**

A total of 90 post-ischemic stroke patients with spastic hemiplegia will be intervened for 4 weeks and followed up for 4 weeks. They will be randomly assigned to three groups including two observation groups and a standard care control group in a 1:1:1 ratio. All patients will receive standard care and regular rehabilitation accordingly. In addition, the two observation groups will receive 12 sessions of Acu-TENS at 20 Hz or 100 Hz for 30 min combined with 1 mA tDCS for 20 min, three times a week, for 4 weeks. The primary outcome is the change in total modified Ashworth scale (MAS) score from baseline to week 4. Secondary outcomes include changes in surface electromyography (SEMG), Fugl-Meyer Motor Function Scale, Modified Barthel Index (MBI), and 10-meter walk test from baseline to week 4. MAS score will also be measured after 4 weeks of follow-up. Adverse events throughout the study will be recorded.

**Discussion:**

This trial will evaluate, for the first time, the therapeutic potentials and safety of Acu-TENS combined with tDCS on spasticity and motor function in stroke patients. It will provide evidence for frequency-dependent anti-spastic effect of Acu-TENS, and a reference for rated parameter setting of new mixed transcutaneous and transcranial stimulation system for stroke rehabilitation, thereby promoting proactive healthcare consequently.

**Trial registration:**

Chinese Clinical Trials Register ChiCTR2200067186.

## Introduction

Stroke has become the main cause of human death and the leading cause of disability worldwide (1, 2), and ischemic stroke patients account for about 70% of the total number of strokes (3, 4). Spastic hemiplegia is characterized by the coexistence of muscle spasms and muscle weakness in one limb, which is the main motor dysfunction after stroke, posing a huge challenge for clinical treatment and patient rehabilitation, with heavy economic burden on healthcare system (5). Significantly, the incidence rate of spastic hemiplegia after stroke can be as high as 39.5% (6). Furthermore, for stroke patients with moderate or severe motor deficits, the increased muscle tone is as high as 97%, significantly reducing the patient’s quality of life (7, 8). Medications such as oral anti-spastic drugs (9), intramuscular botulinum toxin injections (10), and surgery (11) are the most common options for spastic hemiplegia after stroke. Although the effects of muscle relaxants on reducing resistance to passive movement have been confirmed, their uncertain effects on active functional improvement cannot be bypassed (12). Moreover, surgical risks and underlying adverse effects of anti-spastic drugs such as muscle weakness, sensory impairment, drug addiction, and hepatotoxicity (13, 14) should be considered. Additionally, since the prevalence rates of stroke rise significantly with age and is usually associated with poorer results (15), population ageing in many countries, especially in China, might lead to a substantial increase in the elderly stoke patients with complex medical care needs, driving up disease and care burdens (16, 17). Therefore, patients and their families as well as doctors aspire to more effective and safer therapies that can promote functional rehabilitation for the patients.

Emerging data including systematic reviews (18–21) and recent clinical studies (22) have shown that acupuncture and its related therapies, plays an important role in treating the patients with spastic hemiplegia following ischemic stroke. Clinically, using of several acupoints in one treatment session always exert better effects compared with a single point (22) due to the synergistic effect of acupoint combination (23). Applying transcutaneous electrical nerve stimulation (TENS) to stimulate specific acupoints, is a non-invasive acupuncture related therapy known as acupoints-based TENS (Acu-TENS) or transcutaneous electrical acupoints stimulation (TEAS). It is believed that Acu-TENS can induce signal transmission or restore the energy balance of the human body similar to acupuncture (24).

As a widely used noninvasive brain stimulation technique in recent decades, transcranial direct current stimulation (tDCS) via delivering a slight electrical current between two electrodes placed on the scalp, shows potential therapeutic effects for various neurological diseases including post-stoke spasticity (25). Interestingly, in acupuncture, a new developed innovative modality named scalp acupuncture has gained increasing interest for rehabilitation in stroke survivors with satisfied effects (26). However, scalp acupuncture may cause some adverse reactions such as stabbing pain, mild fainting or scalp hematoma (27), which consequently decrease the treatment compliance of patients to a certain degree. Therefore, we hypothesized that applying acupoints-based TENS on limbs combined with tDCS on scalp may perform synergic effect, so as to ameliorate spasticity and improve motor recovery in ischemic stroke survivors with spastic hemiplegia.

Clinical experience indicates that identifying appropriate application sites and stimulation parameters are crucial to the efficacy of any modalities based on acupoints. Evidence of the positive effects of tDCS on post-stroke spasticity has been provided by several studies (28–30), with similar intervention paradigm to stimulate affected motor cortex with a current in 1 mA. Actually, the core stimulation site of scalp acupuncture for post-stroke hemiparesis, named Anterior Oblique Line of Vertex-Temporal, is almost the same location as the surface area of motor cortex on the scalp (31). However, in the studies applying TENS or TEAS to post-stroke spasticity, although the anti-spastic effects are all satisfied, there is a wide range of frequency from 2 to 100 Hz (32–36). Thus, the optimal stimulation frequency of Acu-TENS remains unclear and needs to be furtherly investigated.

Based on the above, the present study aimed to evaluate the therapeutic effects and safety of Acu-TENS combined with tDCS for post-ischemic stroke with spastic hemiplegia. Since 20 and 100 Hz of TENS were the most commonly used frequencies in the majority of corresponding studies (32, 37), we attempt to investigate the optimal frequency of transcutaneous electrical stimulation by comparing the different stimulation frequencies at 20 and 100 Hz of Acu-TENS. Furthermore, it will provide a basis for future clinical trials with larger sample size and a reference for the rated parameter setting of new mixed acupoints-based transcutaneous electrical stimulation devices.

## Design and methods

### Study design

**Figure 1:**
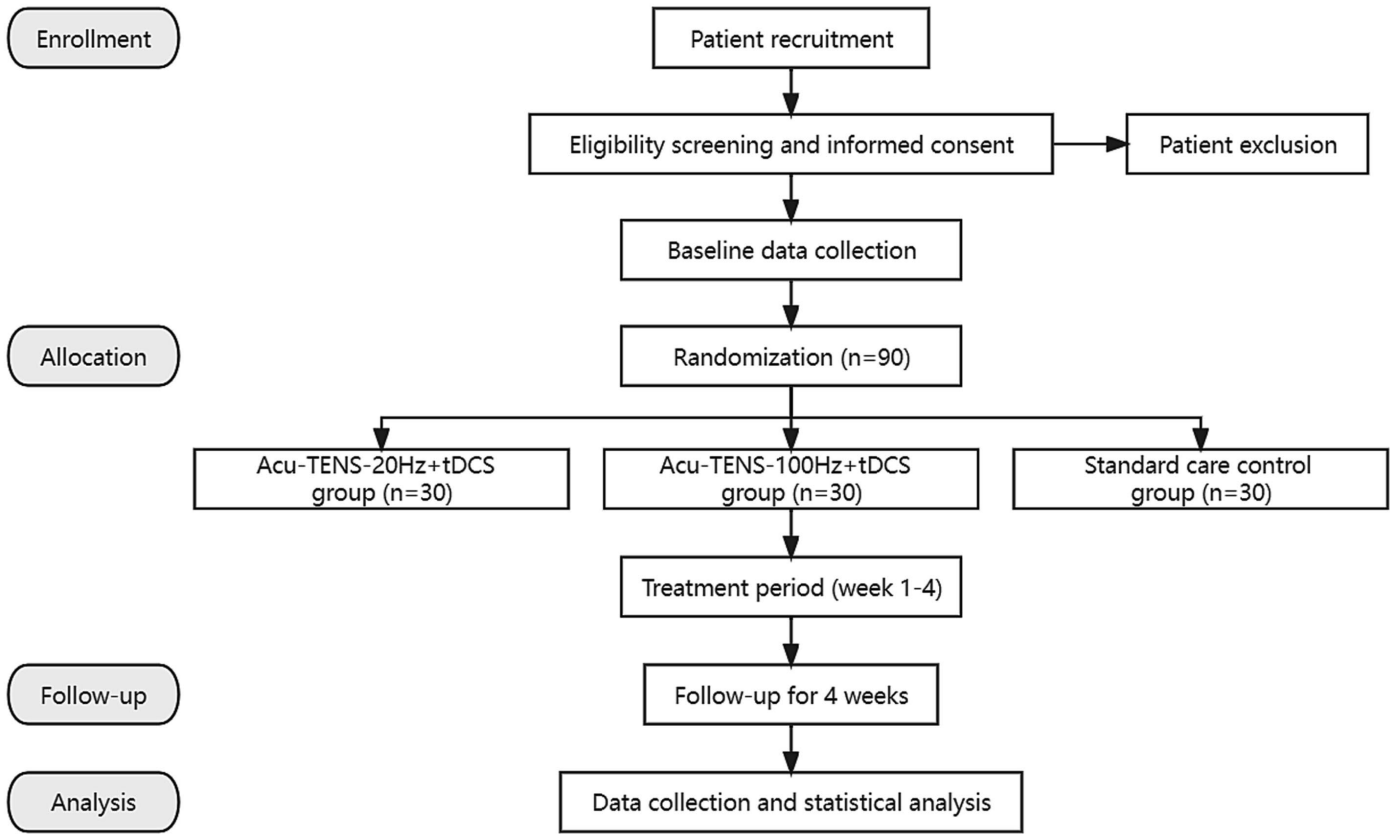
Flow chart of the trial. Acu-TENS, acupoints-based transcutaneous electrical nerve stimulation; tDCS, transcranial direct current stimulation.

This is a prospective, assessor-blinded, randomized, controlled pilot trial with three parallel groups. A total of 90 post-ischemic stroke patients meeting the inclusion criteria will be recruited and then randomly allocated into 3 groups of 30 patients each. All patients will receive 4 weeks of standard care, and the patients assigned to 2 observational groups will receive tDCS and Acu-TENS treatments during a 4-week period (tDCS at 1 mA, Acu-TENS at 20 or 100 Hz) additionally. The stimulation waveform, sites, and duration will be kept the same throughout the study. This study will be performed in conformity to the principles of the Declaration of Helsinki. The “Standard Protocol Items: Recommendations for Interventional Trials” (SPIRIT) will be followed in reporting this protocol and the checklist is provided in Supplementary Material. The study protocol outline is presented as a flow chart in Figure 1, while the study timeline is illustrated in Table 1.

**Table 1:** Time schedule of the trial process.

	**Baseline**		**Treatment**				**Follow-up**
		Week 0	Week 1	Week 2	Week 3	Week 4	Week 8
**Enrollment**							
Eligibility screen	**×**						
Informed consent	**×**						
Random allocation		**×**					
**Interventions**							
Acu-TENS-20 Hz + tDCS			**×**	**×**	**×**	**×**	
Acu-TENS-100 Hz + tDCS			**×**	**×**	**×**	**×**	
**Standard care control**			**×**	**×**	**×**	**×**	
**Evaluations**							
MAS		**×**	**×**	**×**	**×**	**×**	**×**
SEMG		**×**				**×**	
Fugl–Meyer motor function scale		**×**				**×**	
MBI		**×**				**×**	
10-meter walk test		**×**				**×**	
**Adverse events**			**×**	**×**	**×**	**×**	

The intervention will last for 12 sessions, three times a week for 4 weeks. MAS, modified Ashworth scale; SEMG, surface electromyography; MBI, Modified Barthel Index.

### Recruitment

Post-ischemic stroke patients with spastic hemiplegia will be recruited from hospitals in Beijing, China. Posters and flyers will be used to recruit patients. Study information such as the purpose of the study, intervention methods, process and possible adverse reactions will be introduced to the potential participants. Patients will be screened according to inclusion and exclusion criteria by trained researchers. Informed consent will be signed prior to enrollment by patients who meet the requirements.

### Participants

#### Inclusion criteria

Patients meet all of the following criteria will be considered as eligible participants.

1. Patients with ischemic stroke confirmed by CT or MRI, with first onset of stroke, and onset of stroke for more than 1 month.
2. The clinical symptoms include unilateral limb paralysis and motor dysfunction of upper and lower limbs.
3. The modified Ashworth scale (MAS) rating of grade I or above for the major involved joint of the limb.
4. Able to walk independently for 10 meters without help.
5. Aged ≥18- years, regardless of gender.
6. Stable state, clear consciousness, and no hearing and comprehension disabilities to complete the questionnaires.
7. Participate voluntarily and sign an informed consent form.

#### Exclusion criteria

Patients who meet any of the following criteria will be excluded from this study.

1. Patients with serious internal diseases, tumors and other malignant diseases, infectious diseases, and mental illnesses.
2. Contraindications for electrical stimulation devices: such as with pacemakers or other implantable devices; history of epilepsy or/and seizures; history of head trauma or head and spinal cord surgery; bleeding disorders; in pregnancy or lactation or planning to become pregnant in the next 6 months; injuries, swelling, or scarring at the skin of the stimulating sites; sensory disturbance or allergy to electrical stimulation.
3. Have taken muscle relaxant drugs in the past month or have received botulinum toxin injections in a spastic limb within the past 6 months.
4. Have received transcranial direct current stimulation, transcutaneous electrical stimulation or acupuncture in the past 6 months.

#### Withdrawal criteria

Following patients will be withdrawn from the study:

1. Those who undergo serious adverse events.
2. Those with deterioration of disease or have a serious complication during the study.
3. Those who withdraw informed consent for any reason.

### Randomization and blinding

Eligible participants will be randomly assigned to 3 groups in a ratio of 1:1:1 by an independent statistician using a randomization sequence generated with SPSS 25.0 and will be concealed from the investigators. The random number of patients meeting criteria will be telephoned by the unaffiliated investigator. Due to the characteristics of interventions, participants in the standard care control group will be aware of their allocation ineluctably.

An independent researcher will pre-set the frequency of the Acu-TENS and then cover the frequency display site to ensure the operators remain blinded to the allocation (different frequencies of Acu-TENS) throughout the study. To reduce the risk of bias, the evaluators and statisticians will be blinded to group allocation during outcome assessment and data analysis.

### Intervention

**Figure 2:**
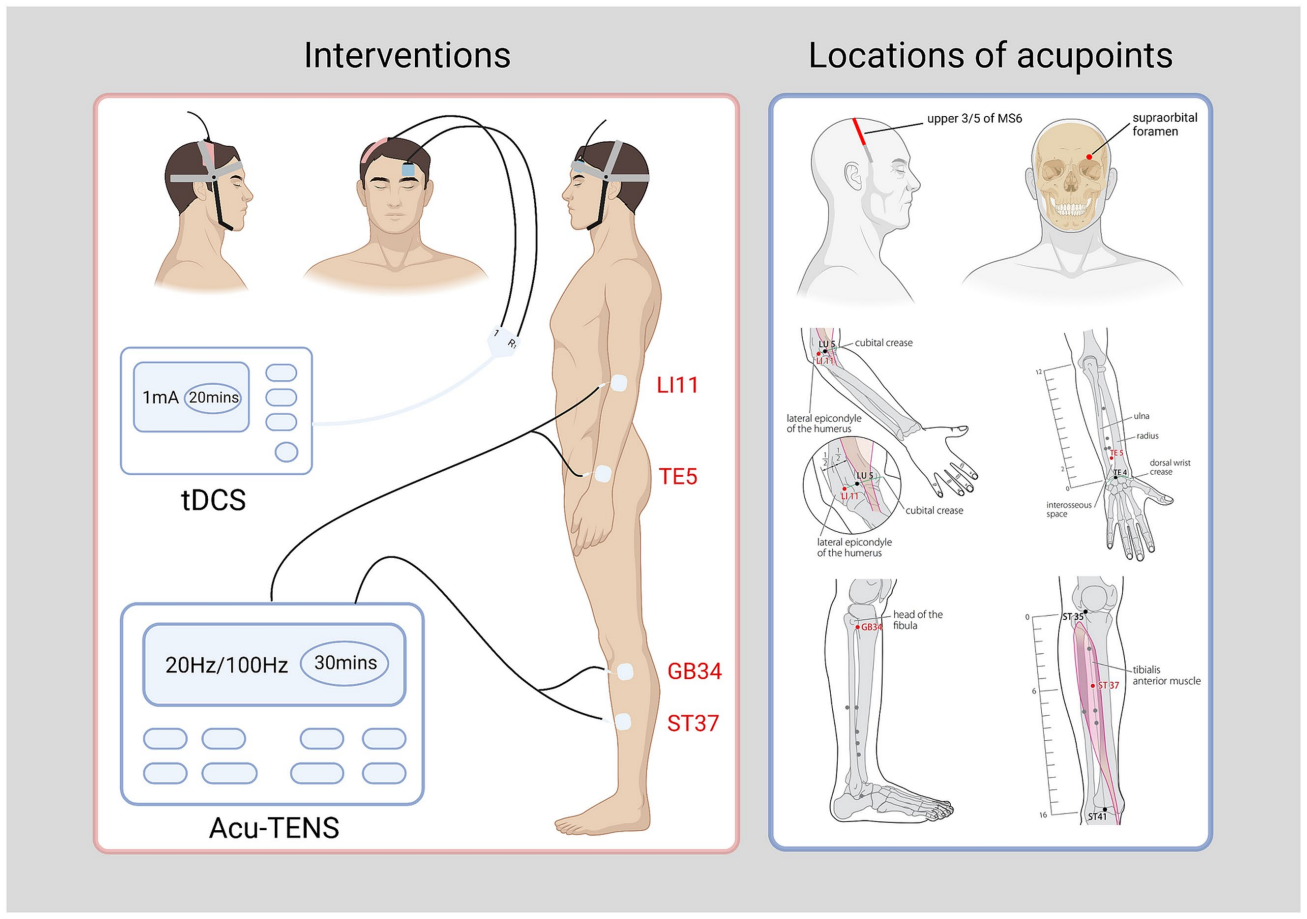
Interventions and locations of acupoint (right head injury and left limbs affected as an example). tDCS, transcranial direct current stimulation; Acu-TENS, acupoints-based transcutaneous electrical nerve stimulation; MS 6, anterior oblique line of vertex-temporal; LI 11, Quchi; TE 5, Waiguan; GB 34, Yanglingquan; ST 37, Shangjuxu. Four acupoints figures of LI 11, TE 5, GB 34, and ST 37 cited from WHO (37, 38). Figure was created on Biorender with permission to publish.

Throughout the trial, all participants will receive standard care in modern medicine for ischemic stroke, corresponding symptomatic or supportive treatment, and regular rehabilitation treatment as well. Simultaneously, Acu-TENS combined with tDCS will be additionally applied to the participants in 2 observational groups. In this study, the acupuncture meridian points used for Acu-TENS and the stimulation locations of International Standard Scalp Acupuncture used for tDCS are referenced from the standards determined by the World Health Organization (38, 39). The locations of the acupoints used in this trial and the methods of electrodes connection are illustrated in Figure 2, while the detailed locations of acupoints are shown in Table 2. All acupoints-based treatment will be conducted independently by the same two acupuncturists, who have at least 5 years of work experience and to be trained specifically in a standard process prior to the study.

**Table 2:** Locations of acupoints [WHO (37, 38)].

**Acupoints**	**Side**	**Locations**
Quchi (LI 11)	Affected limbs side	On the lateral aspect of the elbow, at the midpoint of the line connecting the depression lateral to the biceps brachii tendon with the lateral epicondyle of the humerus.
Waiguan (TE 5)	Affected limbs side	On the posterior aspect of the forearm, midpoint of the interosseous space between the radius and the ulna, 2 *cun* proximal to the dorsal wrist crease.
Yanglingquan (GB 34)	Affected limbs side	On the fibular aspect of the leg, in the depression anterior and distal to the head of the fibula.
Shangjuxu (ST 37)	Affected limbs side	On the anterior aspect of the leg, 6 *cun* inferior to the lateral depression to the patellar ligament of the knee.
Anterior Oblique Line of Vertex-Temporal (MS 6)	the injured side of head (Opposite to the affected limbs)	From the Qianshencong (EX-HN1, at the vertex of the head, 1 *cun* anterior to Baihui GV20) oblique to the Xuanli (GB6).

Cun: a unit used to locate acupuncture points.

#### Observational groups

Patients will receive Acu-TENS at 20 Hz or 100 Hz combined with the same tDCS treatment. The intervention will last for 12 sessions, each 20 min for tDCS and 30 min for Acu-TENS, 3 times a week for 4 weeks. Treatment will be performed using a transcranial electrical stimulator [NeuStim NSS10E, Neuracle Technology (Changzhou) Co., Ltd., Changzhou, China] and a transcutaneous electrical nerve stimulator (QL/T-IIA, Sichuan Qianli Beoka Medical Technology Co., Ltd., Chengdu, China).

The NeuStim NSS10E transcranial electrical stimulator has two output channels, one of which is used to connect a pair of rectangular electrodes (Anode 50 × 100 mm, Cathode 50 × 50 mm). The patient will be placed in a comfortable supine position to fully expose the acupoints on the head and affected limbs. After skin cleaning, the anode will be placed on the upper 3/5 of the Anterior Oblique Line of Vertex-Temporal (MS 6) of scalp acupuncture on the injured side, and the cathode will be placed on the supraorbital region on the contralateral side. The tDCS will applied for 20 min at a current intensity of 1 mA (29, 40).

The QL/T-IIA transcutaneous electrical nerve stimulator has 2 output channels, each connected to a pair of electrodes (40 mm × 40 mm). After skin cleaning, each pair of electrodes will be attached at two acupoints on the affected limbs respectively, Quchi (LI 11) and Waiguan (TE 5) on the upper limb, while Yanglingquan (GB 34) and Shangjuxu (ST 37) on the lower limb in a square wave with a pulse width of 0.2 ms. The frequency will be set to either 20 or 100 Hz depending on the grouping of patients. The stimulation intensity of each output will be adjusted to 2–3 times of the sensory threshold of each participant without eliciting a muscle contraction. The Acu-TENS will last for 30 min.

#### Standard care control group

Participants assigned to the standard care control group will only receive standard management according to the guidelines in modern medicine for ischemic stroke, including symptomatic or supportive treatment, and regular rehabilitation treatment individually, without Acu-TENS or tDCS.

### Outcome measurement

#### Primary outcome

The primary outcome will be the change from baseline in muscle tone assessed with MAS in elbow, wrist, knee, and ankle 4 weeks after baseline (the end of the intervention). The detailed scoring rules of MAS with 6-level (level 0, 1, 1+, 2, 3, 4) are consistent with previous study (41). In the analysis of MAS scores, a derived MAS score with a 6-point scale from 0 to 5 (level 0, 1 equivalent to 0 and 1 respectively, level 1+ transformed into 2, 2 into 3, 3 into 4, and 4 into 5) will be used for practicality. To ensure the objectivity and accuracy of the MAS assessment, the independent evaluator will be instructed via specific training to use a medium velocity of passive movements, lasting about 1 s (42). Subsequently, MAS will be measured for above mentioned four joints with different levels in each joint, and then the sum of MAS scores in four joints will be recorded as the total MAS score.

#### Secondary outcomes

Secondary outcomes will involve changes in surface electromyography (SEMG) of the lower arm and calf muscles on the hemiplegia side, Fugl-Meyer Motor Function Scale, Modified Barthel Index (MBI), and 10-meter walk test among the three groups at different time-points. Assessment of all secondary outcomes will be performed at baseline and at the end of the treatment (week 4).

**Figure 3:**
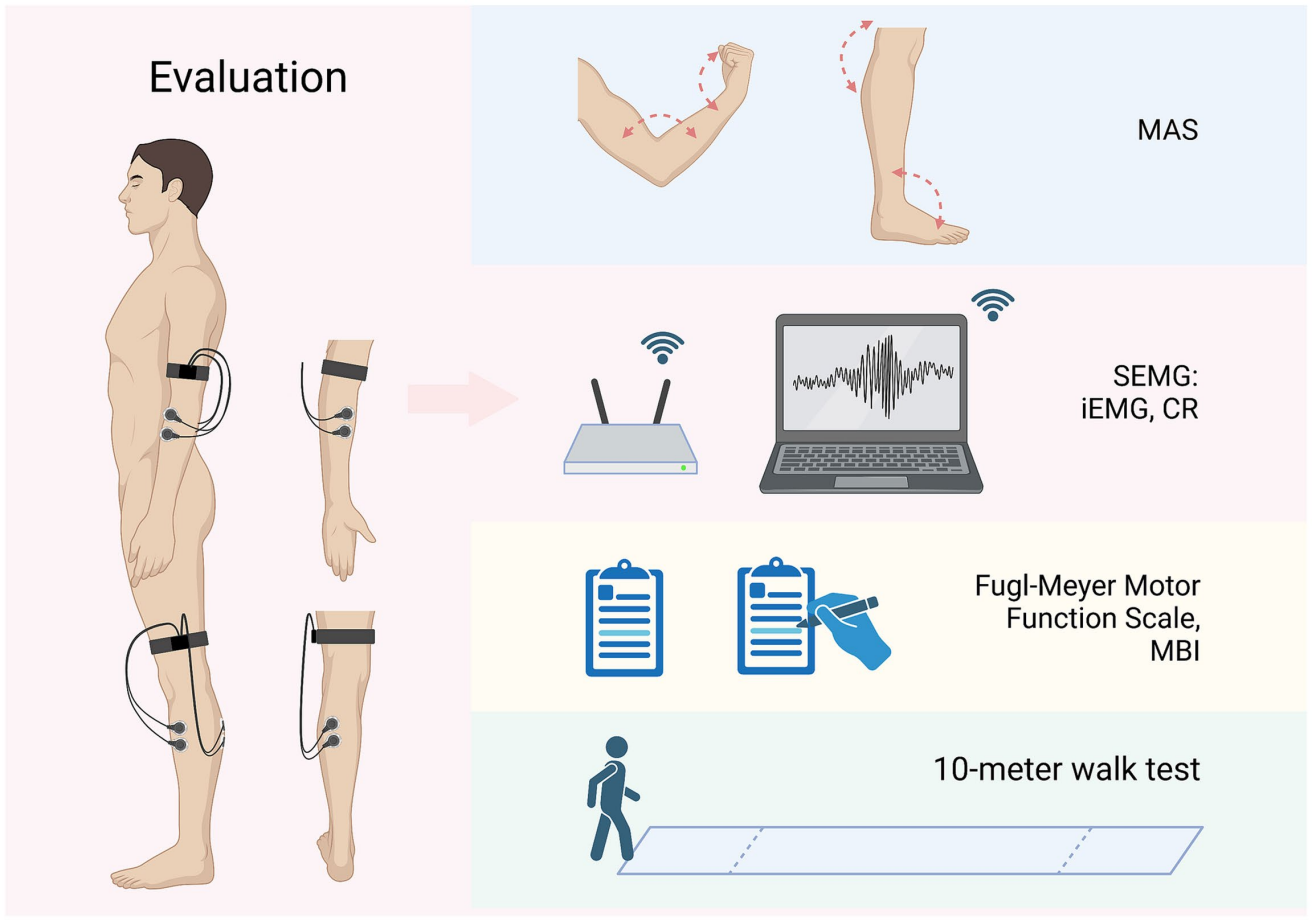
Evaluations and outcomes (left limbs affected as an example). MAS, modified Ashworth scale; SEMG, surface electromyography; iEMG, integrated EMG; CR, co-contraction ratio; MBI, Modified Barthel Index. Figure was created on Biorender with permission to publish.

SEMG can reflect the myoelectric activity of muscles. In this trial, the SEMG results of the flexor carpi radialis muscle, extensor carpi radialis longus muscle, gastrocnemius muscle, and tibialis anterior muscle will be evaluated by an EMG acquisition system [NSW304M, Neuracle Technology (Changzhou) Co., Ltd., Changzhou, China] at baseline and week 4. The myoelectric electrodes will be affixed regarding the procedure recommended by the European Commission (43), using round Ag/AgCl electrode sheets with a diameter of 10 mm, 20 mm apart to the middle of the muscle belly, as shown in Figure 3. The patient will be asked to relax and be quiet for at least 3 min before the assessment. After starting the recording, the EMG of the patient’s quiet state will be first collected for 3 min, and then the patient’s wrist or ankle joint will be rapidly flexed and extended 3 times, with an interval of 1 min each time. The maximum value of 3 repetitions will be taken as the integrated EMG (iEMG) value. The data will be analyzed from the recordings, including iEMG and co-contraction ratio (CR). The CR value can be calculated according to this formula:

CR (%) = iEMG value of antagonistic muscle/(iEMG value of agonistic muscle+ iEMG value of antagonistic muscle) × 100%.

The Fugl–Meyer Motor Function Scale is a 100-point scale consisting of 66 points for the upper extremities and 34 points for the lower extremities, and is a commonly used scale for assessing patients’ motor function (44). The MBI is also a 100-point scale that evaluates the degree of assistance needed for activities of daily living (45). The 10-meter walk test can assess the patient’s lower limb movement. Patients will be advised to complete both questionnaires and the walk test at baseline and week 4. Figure 3 demonstrates the method of above assessments.

### Safety evaluation

The investigators and operators will closely monitor the patients during the 4-week treatment period. If the patient feels any discomfort, the intervention will be stopped immediately, and the patient’s condition will be treated appropriately. Any adverse reaction caused by electrical stimulation, such as localized redness, swelling, pain, or other adverse events, will be recorded by operators.

### Sample size calculation

This study is a small sample clinical trial, and as to be a preliminary for the further large sample clinical trial. Based on a previous study (46), PASS 15.0 software (NCSS Statistical Software, Kaysville, UT, USA) was used to determine the sample size, and by setting the MAS scores decreased in the three groups by 1, 0.75, and 0.45, respectively, with a standard deviation of 0.52, a two-sided *α* = 0.05 with 90% power, the minimum sample size was 24 cases per group. Considering a 20% dropout rate, 30 patients in each group and a total of 90 patients in three groups were needed.

### Data management

The data required for the study of all patients will be recorded on the printed case report forms (CRFs), including observation time points, outcome measures, adverse reactions, and safety evaluations. The private information of each participant, including name, phone number and other identity will be protected from disclosure. An independent data manager will check the accuracy and timeliness of the data and keep all data safe. No artificial data modification will be allowed after the end of the trial.

### Quality control

To ensure the high quality of the trial, all investigators and operators will be trained uniformly and professionally before the start of the trial. The training includes understanding the trial protocol and process and clarifying the tasks of each recruitment, treatment and evaluation step. During the implementation of the trial, the outcome data and safety will be reviewed and supervised by experts from the Ethics Committee of the Beijing University of Chinese Medicine.

### Statistical analysis

Statistical analyses will be performed by statisticians who are independent of the research team and have no knowledge of the study groupings or study settings. Statistical analyses will be performed using IBM SPSS Statistics 25.0 software and follow intention-to-treat (ITT) principles to ensure all subjects are included after randomization. Missing data will be handled by multiple imputations. Continuous variables that follow a normal distribution are presented as mean ± standard deviation, and those that do not follow a normal distribution are given as median and interquartile range. Comparisons within groups will be performed via paired t-tests, and difference among groups will be analyzed by the one-way ANOVA test. For measurements at different time points, differences were assessed using repeated-measures ANOVA followed by Bonferroni *post hoc* tests. The test level is 0.05, with *p* < 0.05 indicating a statistically significant difference.

## Discussion

The majority of stroke survivors have sequelae including spasticity and motor disorder (47), which might severely weaken the self-care ability of patients, burden the families and public healthcare system. Relieving limb spasticity effectively is crucial to promote motor recovery in spastic hemiplegia after ischemic stroke (48). Increasing evidence showed that acupuncture and noninvasive acupuncture related therapies such as Acu-TENS are widely used for post-stroke spasticity with satisfied results (34, 35, 49). Meanwhile, tDCS also showed positive effects for post-stoke spasticity (28–30). However, to the best of our knowledge, in addition to investigating the effectiveness and safety of Acu-TENS on spasticity and motor function after stroke, few studies have screened the optimal frequency of TENS. Moreover, studies that include both TENS and tDCS based on classical acupuncture points are even more rare. This clinical trial is designed to focus on the optimal stimulation frequency of Acu-TENS in the treatment of post-stroke spastic hemiplegia, to preliminarily evaluate the efficacy and safety of Acu-TENS combined with tDCS therapy, and to offer physicians and patients an additional treatment option. The results will provide a reference for the setting of rated parameter of related mixed transcutaneous and transcranial stimulation system for stroke rehabilitation.

### Elucidation of optimal stimulation parameters for Acu-TENS is essential for its effectiveness

Notably, spasticity rarely tends to resolve on its own but progressively worsens over time (50). Spasticity can exacerbate muscle weakness, leading to severe dyskinesia and further exacerbation of daily activities (51). Therefore, adequate and effective control of post-stroke spasticity is the key to break this vicious cycle, thereby fully facilitating motor recovery (48). Compared to medications and surgery, Acu-TENS therapy is a non-pharmaceutical and non-invasive modality that is more acceptable to patients.

The frequency used in an electrical stimulation therapy always plays a crucial role for its therapeutic effect. The frequency of Acu-TENS is worthy of exploring and researching, since it is used in variety, ranging from 2 to 100 Hz. Two Meta-analysis studies have shown that the frequency of TENS for post-ischemic stroke with spastic hemiplegia is mostly at 100 Hz (32, 33). TENS at 20 Hz has also been used in some studies, with positive effects of improving electromyography, relieving spasticity, and increasing walking speed (37, 52, 53). A very few studies compared the anti-spastic effects of TENS or TEAS at different frequencies in stoke patients. Only one study (36) investigated the effects of both 2 and 100 Hz of TEAS on muscle spasticity following brain injury, the results suggested that both low and high frequencies had anti-spastic effects while 100 Hz was better. Taken together, in this study, we will compare the effects of Acu-TENS at 20 and 100 Hz in treating post-ischemic stroke with spastic hemiplegia to explore the optimal Acu-TENS stimulation frequency. The results of this study could provide a reference for setting optimal rated parameters of noninvasive acupoint-based transcutaneous stimulator, which can be manipulated by patients themselves. This modulation may be an alternative option for some chronic diseases such as post-stroke sequelae, thereby promoting proactive rehabilitation of the elderly.

### Appropriate combination of acupoints may increase the anti-spastic effects of Acu-TENS

According to the classical theory of acupuncture, a formula of acupoints composed by the points with systemic or local regulation effects usually achieve better clinical results due to their synergistic effects (23, 54). In this study, we will choose Quchi (LI11), Waiguan (TE5), Yanglingquan (GB34), and Shangjuxu (ST37), which are located on the antagonistic muscles of upper and lower limbs respectively, as the stimulation points of Acu-TENS (21). Applying Acu-TENS on those four acupoints can better excite the antagonistic muscles, coordinate and balance the muscle tension of the active and antagonistic muscles, relieve spasticity, promote the transition from co-movement to separation movement, hence establish a normal movement pattern finally. Meanwhile, in terms of Traditional Chinese Medicine (TCM) theory, stimulating those acupoints has the effects of promoting the flow of the energy and balancing yin (medial side of the limbs) and yang (lateral side of the limbs) meridians, leading to functional recovery of the muscles and sinews. Recent study indicated that peripheral stimulation such as acupuncture, might produce both local spinal segmental reflexes and supra-spinal reflexes (55). Yanglingquan (GB34), also known as “confluential point of sinews,” has the effect of relaxing and strengthening sinews, thus it is considered as a classic acupoint for relieving spasticity. A fMRI study illustrated that acupuncture at GB34 may improve the motor function recovery in post-stroke hemiplegic patients via affecting the functional connectivity of the brain (56). It was found that the anti-spastic action of acupuncture at GB34 for post-stroke spasticity was related to its alleviation of spinal hyperreflexia via KCC2-mediated spinal GABA_A_ activation (57).

### Combination of Acu-TENS and tDCS might be an option for stroke rehabilitation

After a stroke, the body’s central nervous system immediately starts the stress repair function, which often leads to abnormal neuroplasticity and spasticity simultaneously (58). Although spastic hemiplegia is mainly manifested by contracture of limb muscles and joints, the root cause of spasticity lies in impaired brain function. Therefore, in terms of TCM, “co-morbidity of body and mind” can be considered as the basic clinical characteristic of spastic hemiplegia after stroke, while any modality based on the principle of “co-regulation of body and mind” should has certain clinical significance. Using TENS (34, 35) or tDCS (28, 30) alone has been proven effective for post-stroke spastic hemiplegia. A recent review (59) suggests that the combination of transcranial stimulation and peripheral transcutaneous stimulation techniques may have a synergic effect to optimize neuroplastic changes and improve motor recovery after stroke. Such an effect might be that tDCS depolarizes neuronal membrane potential and modulates GABA, Glutamate levels (60) and BDNF (brain-derived neurotrophic factor) expression levels in localized brain regions (61), whereas Acu-TENS activates KCC2-GABA_A_ pathway (57). Therefore, this synergic action of TENS combined with tDCS based on acupuncture points on post-stroke spastic hemiplegia needs to investigate further in clinical trials.

In this study, the anode of tDCS is placed on the upper 3/5 of the Anterior Oblique Line of Vertex-Temporal (MS6) of scalp acupuncture on the injured side. The location of MS6 is a projection of the motor cortex on the scalp, whose upper 1/5 corresponds to the lower limbs and trunk, and the middle 2/5 corresponds to the upper limbs. It has a good therapeutic effect on patients with post-stroke motor disorders and the locations of scalp acupuncture can also be applied to other neuromodulation modalities including tDCS (62). In addition, tDCS stimulation in stroke patients typically lasts for 20 min and uses frequencies of either 1 or 2 mA (63). However, research has shown that 2 mA stimulation poses a higher risk for adverse effects compared to 1 mA stimulation (64, 65). This study opted for the safer and more effective option of using a continuous 1 mA stimulation for a duration of 20 min. Thus, the results of this study are expected to provide new evidence of the effectiveness and safety of using both Acu-TENS and tDCS for post-stroke spastic hemiplegia.

### Multi-perspective assessment on spasticity and motor function is necessary to evaluate the efficacy of modalities for stroke rehabilitation

To evaluate the efficacy of a treatment modality with more reliable evidence has been a challenge in many clinical trials. Even in some high quality studies with rigorous methodology, lack of objective outcomes is one of the most common limitations (66, 67). In this study, we attempt to observe the effects of combination of Acu-TENS and tDCS on spasticity and motor function in stroke patients from multiple perspectives. Various functional assessments for spasticity and motor function including MAS, Fugl–Meyer Motor Function Scale, MBI, and 10-meter walk test will be used. In addition to subjective measures, we will also detect the iEMG and CR value of SEMG of spastic and antagonistic muscles, as an objective assessment. The SEMG is a non-invasive and painless method for muscle function assessment, which is widely used in rehabilitation researches as an objective and reliable quantitative outcome measure (68, 69). Commonly used signal evaluation indexes of SEMG include frequency and time domain indexes, of which iEMG value is a time domain index. The iEMG value can respond to the total amount of discharges of the skeletal muscles involved in a unit of time, reflecting the contraction characteristics of the muscles in a unit of time, and its value is positively correlated with the muscle strength and muscle tension to a certain extent (70). Normal joint movement requires the coordinated contraction of active and antagonist muscles, and the CR value reflects the proportion of muscle activation of antagonist muscles during active muscle contraction. It has been shown that the co-contraction rate of antagonist muscles is significantly increased in stroke patients compared to healthy controls. SEMG is considered an objective and credible method to assess the muscle tone of spastic and antagonistic muscles. Therefore, we will combine the subjective and objective measures in this study, expecting for providing more reliable evidence of a new modality of Acu-TENS combined with tDCS for stroke rehabilitation.

## Limitations

First, not all patients can be blinded. The sham Acu-TENS and tDCS will not be applied to the standard care control group, which may lead to psychological influence for patients in this group to a certain degree. We will also ensure that patients in both observation groups are blinded to the specific stimulation frequency. Second, although 4 and 8 weeks are clinically standard treatment and follow-up periods for the commonly used modalities based on acupoints, future studies may require more extended periods to observe the long-term effects of Acu-TENS combined with tDCS in ischemic stroke patients with spastic hemiplegia. Third, although MAS is the most commonly used clinical scale for evaluating spasticity in the majority of studies, the results of muscle tone assessed with MAS remained unavoidably subjective. Fourth, since the elderly are a group of higher risk of stroke, the inclusion criteria does not limit recruitment to patients over the age of 60 in present study. In future study, the elderly can be a group of interest for recruitment. Finally, to confirm the results of this study, further expansion of the sample size is needed in future studies.

## Ethics statement

The protocol has been approved by the Ethics Committee of the Beijing University of Chinese Medicine (No. 2022BZYLL1208) as well as registered with the China Clinical Trials Registry (ChiCTR2200067186). The participants provided written informed consent to participate in this study.

## Author contributions

XQ: Conceptualization, Writing – original draft. L-XM: Conceptualization, Writing – review & editing. L-HM: Writing – review & editing, Software. Q-YZ: Writing – review & editing. J-YX: Writing – review & editing. X-YW: Writing – review & editing. T-YS: Writing – review & editing.
